# Genome-Wide Analysis of Barley bHLH Transcription Factors and the Functional Characterization of *HvbHLH56* in Low Nitrogen Tolerance in Arabidopsis

**DOI:** 10.3390/ijms24119740

**Published:** 2023-06-04

**Authors:** Xiaoyan Quan, Chen Meng, Ning Zhang, Xiaoli Liang, Jialin Li, Hongmei Li, Wenxing He

**Affiliations:** School of Biological Science and Technology, University of Jinan, Jinan 250022, China

**Keywords:** genome-wide analysis, bHLH transcription factor, barley, low nitrogen stress, expression pattern

## Abstract

Improvement of low nitrogen (LN) tolerance or nitrogen use efficiency (NUE) in crops is imperative for environment-friendly agriculture development. The basic helix-loop-helix (bHLH) transcription factors are involved in multiple abiotic stresses and are suitable as candidate genes for improving LN tolerance. Few studies were performed on the characterization of the *HvbHLH* gene family and their function in response to LN stress in barley. In this study, 103 *HvbHLH* genes were identified through genome-wide analysis. HvbHLH proteins were classified into 20 subfamilies based on phylogenetic analysis in barley, which was supported by conserved motifs and gene structure analysis. The stress-related *cis*-element analysis in the promoters showed that *HvbHLHs* are probably involved in multiple stress responses. By phylogenetic analysis of *HvbHLHs* and bHLHs in other plants, some *HvbHLHs* were predicted to play roles in response to nutrition deficiency stress. Furthermore, at least 16 *HvbHLHs* were differentially expressed in two barley genotypes differing in LN tolerance under LN stress. Finally, overexpression of *HvbHLH56* enhanced LN stress tolerance in transgenic Arabidopsis, suggesting it is an important regulator in LN stress response. The differentially expressed *HvbHLHs* identified herein may be valuable for the breeding of barley cultivars with LN tolerance.

## 1. Introduction

Transcription factors (TFs) play important roles in the growth and development of plants and animals and their responses to the external environment by regulating downstream gene expression. As one of the most critical factors regulating gene expression, TFs have been intensively studied in the field of biological research. Among various TF families, the basic-helix-loop-helix (bHLH) TFs constitute one of the largest TF families [1]. The bHLH proteins are characterized by a conserved bHLH domain consisting of about 60 amino acids. Each domain has two functional regions, i.e., the basic region at the N-terminus and the HLH region at the C-terminus. The basic region comprises about 15 amino acids, including six basic residues, and has DNA-binding activity [2]. The HLH region, containing about 50 amino acids, is composed of two α-helices separated by a variable loop, and it participates in the formation of homodimers or heterodimers [3].

The bHLH TFs were first identified and characterized in mammals and then gradually discovered in other eukaryotic species. At present, genome-wide comprehensive analyses of plant bHLH proteins have been carried out in numerous species [4]. At least 162 and 167 bHLHs have been identified in model plants, Arabidopsis and rice, respectively [5,6,7]. Recently, 225, 208, 155, 152, 124, 142, and 188 bHLHs were identified in wheat, maize, bean, tomato, potato, cucumber, and apple, respectively [4,8,9,10,11,12,13]. However, little similar research has been carried out in barley, although barley is an important crop worldwide.

TFs are excellent candidate genes for developing cultivars with improved tolerance to adverse stress [14,15,16]. In plants, bHLH TFs are involved in many biological processes, such as the regulation of flavonoid biosynthesis, morphology, and the accumulation of fruit pigments [17,18]. In recent years, more and more studies have confirmed that plant bHLH TFs play important regulatory roles in abiotic stress responses such as drought, salt, cold, and nutrition stress [19]. For example, some bHLH TFs confer drought tolerance by regulating stomatal development, photosynthesis, and growth [20] or by promoting root development and abscisic acid synthesis [21]. Some could enhance plant tolerance to salt [22], cold [23], or manganese [24] stress through their overexpression. At present, there have been many studies about the regulatory role of plant bHLH genes in response to iron deficiency stress. For example, overexpression of *PYE*, *AtbHLH115*, *AtbHLH34,* and *AtbHLH104* enhanced iron deficiency tolerance in Arabidopsis, respectively [25,26,27]. Double overexpression of two bHLH genes, *GmbHLH57* and *GmbHLH300*, can significantly improve the iron deficiency tolerance of soybeans [28]. Several studies have also reported that plant bHLHs play a role in low phosphorus tolerance. For instance, the overexpression of *OsPTF1* can significantly increase the tiller ability, root and shoot biomass, and phosphorus content of rice, thereby enhancing its low phosphorus tolerance [29]. In recent years, the study of plant bHLHs in response to low nitrogen (LN) has also been gradually carried out. Jia et al. (2022) found that *NRI1*, a bHLH gene in *Chlamydomonas reinhardtii*, played an important role in regulating the expression of genes responding to nitrogen (N) deficiency [30]. Yang et al. (2016) reported that overexpression of *TabHLH1* can improve the tolerance of N deficiency by increasing the expression of nitrate transporters and the activity of antioxidant enzymes in tobacco [31]. Sanagi et al. (2021) found that FBH4, a bHLH transcription factor, was also independently involved in regulating the induction of genes related to N recycling and reuse under LN stress [32].

Environmental pollution caused by the excessive application of N fertilizers became a big issue. One of the efficient solutions is to develop cultivars with high N use efficiency /LN tolerance. It has been reported that bHLH could improve plant tolerance to N deficiency [30,31,32]. Thus, bHLH genes may be used as suitable candidates for the genetic improvement of plant LN tolerance/high N use efficiency. However, up to date, little has been known about the function of plant bHLH proteins in LN tolerance. In this study, bHLH family genes in barley were identified and characterized at the genome-wide level. In our previous study, RNA-seq analysis showed that *HvbHLH* genes were differentially expressed at the transcription level in the two barley genotypes differing in LN tolerance (XZ149, LN-tolerant, and XZ56, LN-sensitive) [33,34] under LN stress [35]. The major objectives of this study were to determine the characteristics of the *HvbHLH* gene family, explore the differences in *HvbHLH* gene expression between the two contrasting barley genotypes in response to LN stress, and identify the *HvbHLH* genes useful for LN tolerance breeding.

## 2. Results

### 2.1. Identification of bHLH Family Genes in Barley

Members of the *HvbHLH* family were identified by searching against the whole barley genome with the protein sequence of 329 Arabidopsis and rice bHLHs and Hidden Markov Model (HMM) profiles of the bHLH domain (PF00010) using BlastP and the HMMER program [36,37,38], respectively. The bHLH domain was confirmed through NCBI Conserved Domains and Pfam [39,40]. Totally, 190 putative members containing the bHLH domain were obtained in barley after eliminating the redundant protein sequences. Following that, short and incomplete protein sequences were manually removed. Finally, 103 *HvbHLH* genes were identified in the barley genome (Tables S1 and S2).

The physiochemical properties for the 103 typical *HvbHLH* genes, including the gene ID, chromosomal locations, amino acid length, molecular weight (MW), theoretical isoelectric point (pI), and predicted subcellular localizations, are presented in Table S1. The amino acid lengths of *HvbHLH* proteins ranged from 85 (HORVU4Hr1G075340) to 888 (HORVU4Hr1G020740), with MW ranging from 9.63 (HORVU4Hr1G075340) to 96.10 (HORVU4Hr1G020740) kDa. The theoretical pI varied greatly, from 4.63 (HORVU6Hr1G012730 and HORVU6Hr1G012760) to 11.68 (HORVU5Hr1G065450). Most *HvbHLHs* were localized to the nucleus, while seven were localized to other locations such as mitochondria, chloroplasts, and cytoplasm (Table S1) through WoLF PSORT prediction [41].

### 2.2. Multiple Sequence Alignment of HvbHLHs

**Figure 1:**
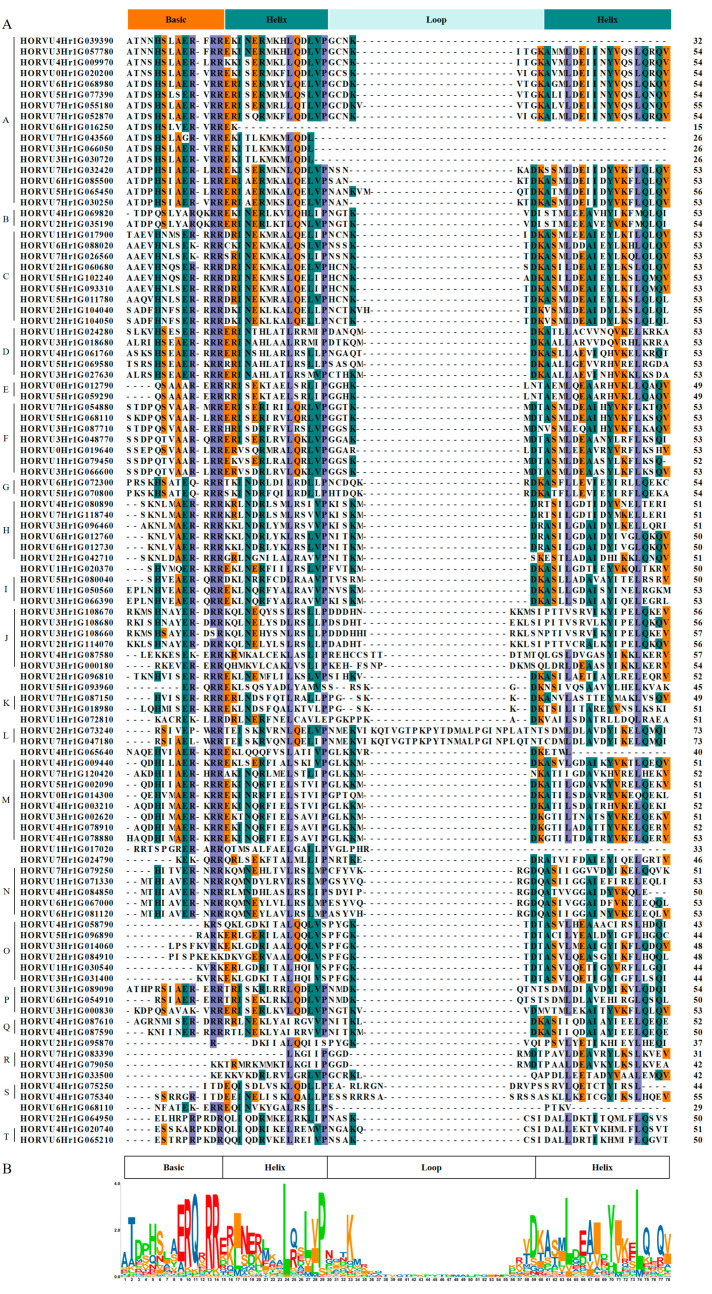
Multiple sequence alignments of the bHLH domains in the HvbHLH proteins. (**A**) Multiple sequence alignment of HvbHLH proteins. Amino acids with greater than 75% identity are shown in purple, those with 50 to 75% identity are shown in green, and those with 35 to 50% identity are shown in orange. Dotted lines indicate gaps. The bHLH domains were labeled. (**B**) Sequence logos of *HvbHLH* domains. The height of each stack represented the conservation of the sequence at that position.

To characterize the features of HvbHLHs, a multiple sequence alignment analysis based on the 103 HvbHLH protein sequences was performed. The conserved amino acids with identity greater than 50% in bHLH domains are labeled with green and purple boxes (Figure 1A). Sequence logos were analyzed employing the 103 HvbHLH domain amino acid sequences (Figure 1B). Four typical bHLH regions—one basic region, one loop region, and two helix regions—were observed in HvbHLH proteins (Figure 1). Most of HvbHLHs contained the complete bHLH domain, except for the absence of the basic region in HORVU7Hr1G083390, the second helix in HORVU1Hr1G017020, and the loop region and second helix in HORVU6Hr1G016250, HORVU7Hr1G043560, HORVU3Hr1G066050, and HORVU3Hr1G030720 (Figure 1A). The number and sequence of the amino acids from the basic region and the two helixes were highly conserved compared with those from the loop region in HvbHLHs. There were two HvbHLH proteins (HORVU2Hr1G073240 and HORVU7Hr1G047180) that had much longer loop regions (31 amino acids), while the other HvbHLH only contained 7–13 amino acids in the loop, which suggests that there may be different regulatory mechanisms existing in the two members [9]. A total of 22 highly conserved amino acids (more than 50% identity) were present in the HvbHLH domain (Figure 1A), which were also observed in the bHLH domain from wheat and *Brachypodium distachyon* [8,42]. Among them, the key amino acid residues Arg-13, Arg-14, Leu-24, Pro-29, and Leu-74 were highly conserved (92.16%, 90.10%, 100%, 90.91%, and 96.84%, respectively) in the 103 HvbHLH proteins (Figure 1B).

### 2.3. Motifs and Exon/Intron Structures Analysis of HvbHLHs

**Figure 2:**
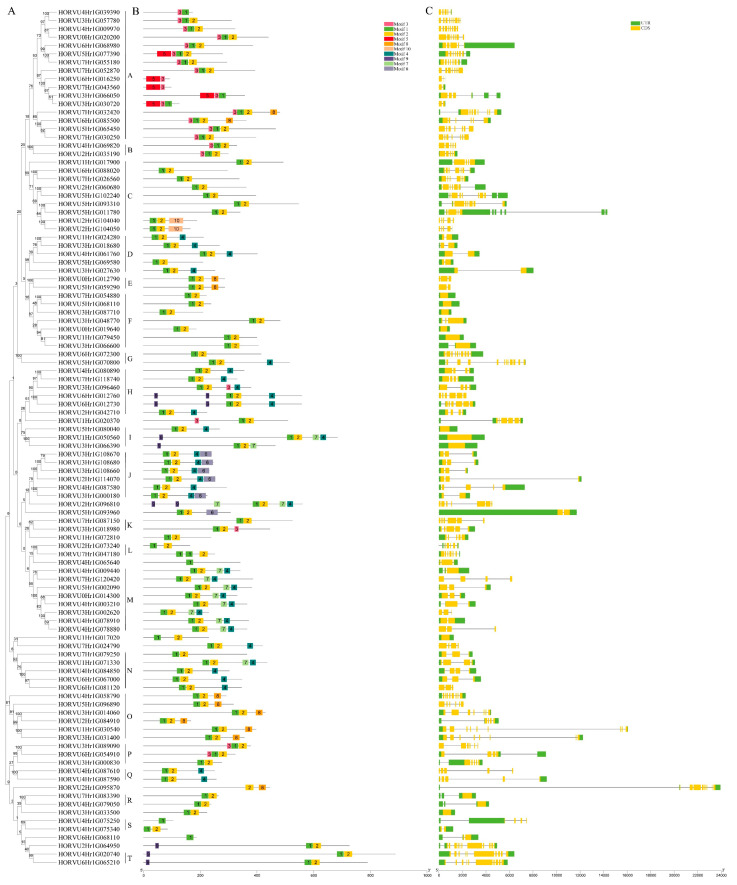
Phylogenetic tree, conserved motifs, and gene structure of the bHLH genes from barley. (**A**) Phylogenetic tree of 103 HvbHLH proteins. The unrooted neighbor-joining phylogenetic tree was constructed with MEGA10.1.8 using full-length amino acid sequences of 103 HvbHLH proteins, and the bootstrap test replicate was set to 1000 times. (**B**) The motif composition of HvbHLH proteins. The motifs, numbers 1–10, are displayed in different colored boxes. The sequence information for each motif is shown in Figure S1. The length of protein can be estimated using the scale at the bottom. (**C**) Exon/intron structure of *HvbHLH* genes. Yellow boxes represent exons, and black lines represent introns. The upstream/downstream regions of *HvbHLH* genes are indicated in green boxes. The length of exons can be inferred from the scale at the bottom.

A phylogenetic tree was constructed on the 103 HvbHLH proteins using the neighbor-joining (NJ) method with bootstrap analysis (1000 replicates). HvbHLH proteins were grouped into 20 subfamilies (A–T) based on the clades with over 50% bootstrap support (Figure 2A). Furthermore, the alignment of genome and coding sequences, as well as exon and intron structure within the coding sequence in bHLH genes, was performed using TBtools [43]. The result showed that most members of the same subfamily had a similar exon/intron structure (Figure 2A,C). For instance, the members in the subfamilies D, I, and R had a relatively simple structure, possessing only 1–2 exons, while the members in the subfamily G had 10–12 exons and were much more complex in their structure.

To investigate the specific motifs of HvbHLH proteins, ten conserved motifs containing 10 to 50 amino acids were predicted (Figure S1) through MEME 5.5.1 software [44]. Almost all of them contained two adjacent conserved motifs, motifs 1 and 2. Motif 1 was composed of basic regions and the first helix, while motif 2 comprised a loop and the second helix. The gaps between these two motifs varied depending on their loop lengths. For instance, the two HvbHLH proteins with a longer loop region we mentioned above (HORVU2Hr1G073240 and HORVU7Hr1G047180) exhibited a larger gap between motifs 1 and 2. HvbHLH proteins consist of 1–5 motifs, and those in the same subfamily exhibited a similar motif composition (Figure 2B). For example, motifs 1, 2, 4, and 7 were identified in all eight members of subfamily M, and motifs 1, 2, and 8 were in all six members of subfamily O. Furthermore, some motifs are unique to one or more subfamilies. For instance, motif 5 only existed in subfamily A, motif 6 was specifically shared by each member in subfamily J, and motif 10 was only found in subfamily C (Figure 2A,B). This conservation of the motif composition patterns in each subfamily might indicate their similar biological functions in the same subfamily [45].

### 2.4. Chromosomal Distribution and Gene Duplication of HvbHLH Genes

**Figure 3:**
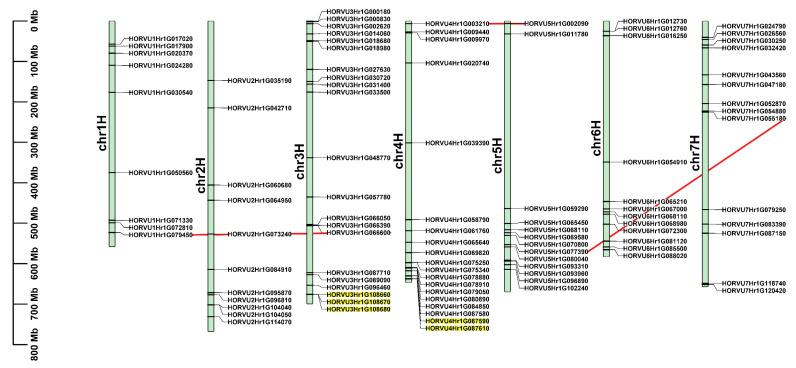
Chromosomal distribution and duplication events for bHLH genes in barley. The tandem duplicated genes are represented by yellow rectangles, and the segmental duplicated genes are linked by red lines. The chromosomes are numbered between 1 and 7 and shown at the left of each chromosome. The scale bar on the left indicated the length (Mb) of barley chromosomes.

According to their physical positions, 99 *HvbHLH* genes were nonrandomly mapped on seven barley chromosomes, with most located at both ends of the chromosomes. More than one-third of the *HvbHLH* genes were located on Chr3H (21 members) and Chr4H (19 members), and only nine genes were located on Chr1H (Figure 3). In addition, four *HvbHLHs* were distributed on scaffolds (Chr0H) (Table S1).

Gene duplication is an important event for the evolution of the plant genome, leading to the generation of new genes [46]. To determine the duplication of *HvbHLHs*, the syntenic regions were analyzed. Totally, 899 segmental duplication blocks and 1913 tandem duplication gene pairs were found in the barley genome (Table S3). There were at least three segmental duplication gene pairs in the *HvbHLH* family (Figure 3 and Figure S2). In addition, three and two tandem duplicated *HvbHLH* genes were observed in Chr3H and Chr4H, respectively (Figure 3 and Figure S2). It is noticed that HORVU4Hr1G087580 is next to HORVU4Hr1G087590 on Chr4H, but it is acceptable to exclude it as a tandem duplicated gene as they are in different subfamilies. Each pair of duplicated genes shared a similar intron-exon structure and motif composition (Figure 2 and Figure 3), suggesting that gene duplication contributed to the expansion of the *HvbHLH* family during the evolution of the barley genome [9].

### 2.5. Function Prediction of HvbHLHs Based on Phylogenetic Analysis

**Figure 4:**
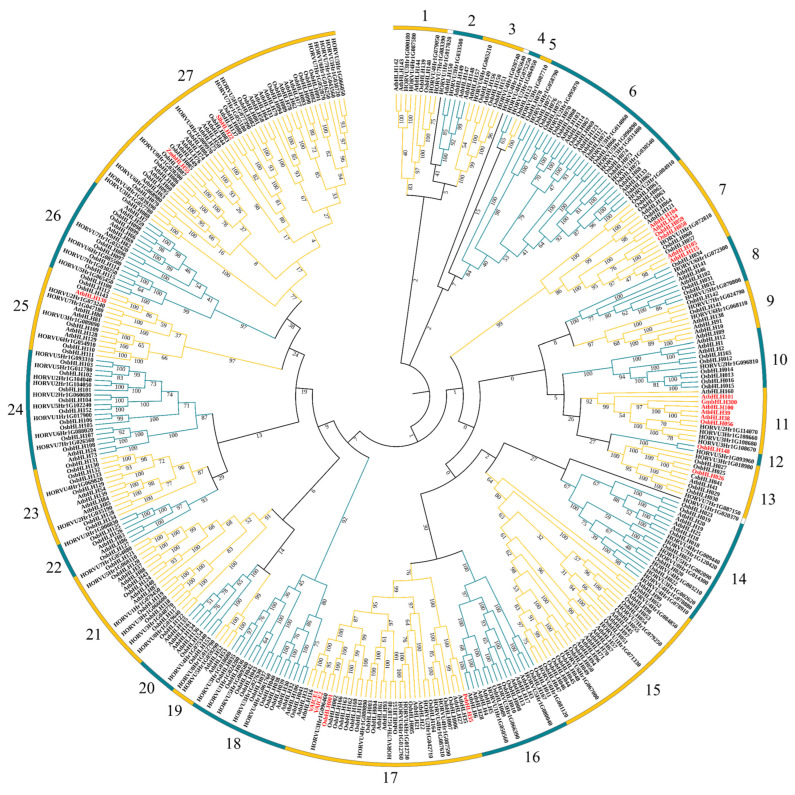
Phylogenetic analysis of bHLH proteins among different species. A phylogenetic tree was constructed using MEGA10.1.8 with full-length amino acid sequences of bHLHs from barley, *Arabidopsis thaliana*, rice, and those related to stress response in other species, including *VaICE1* (KC815984.1), *VaICE2* (KC815985.1), *PebHLH35* (KJ363186.1), *ZmbHLH55* (AIB04349.1), *SlbHLH22* (Solyc03g097820), *CsbHLH041* (XP_011659992.1), *GmbHLH300* (NP_001354033.1), and the bootstrap test replicate was set as 1000 times. Sequence accessions of proteins known to be associated with the stress response have been highlighted in red. The different-colored arcs indicate different subgroups of the bHLHs.

In order to analyze the evolutionary relationship of the *HvbHLH* genes within the bHLH family, the phylogenetic tree was constructed using 103 *HvbHLHs* in barley, 115 *AtbHLHs* in Arabidopsis, and 149 *OsbHLHs* in rice, coupled with 7 *bHLHs* known to be involved in abiotic stress responses in other plants [19]. The result showed that these 374 bHLH proteins were divided into 27 subgroups (Subgroup 1 to 27, Figure 4) based on the clades with over 50% bootstrap support and the classification of bHLH proteins in Arabidopsis and rice [6,7]. Subgroups 4, 5, and 12 were the smallest, each with two members. Subgroup 27 was the largest, having 12, 17, 20, and 2 members in barley, rice, Arabidopsis, and other plants, respectively.

The functions of HvbHLHs were predicted in accordance with the verified functional homologs in the same subgroup. Some bHLH proteins of subgroups 12, 13, 17, and 27 were involved in different abiotic stress responses, such as drought [12,47], cold [48], and salt [49]. In addition, many proteins in subgroups 7, 11, and 25 respond to nutrition stress, such as iron deficiency [19] and low nitrogen stress [32]. The predicted functions of the HvbHLH are present in Table S1. In general, there will be some reference value for follow-up function research, although the functions of HvbHLHs could not be clearly deciphered by the evolutionary relationships.

### 2.6. Stress-Related Cis-Elements in the Promoter of HvbHLH Genes

To understand the potential regulatory patterns of *HvbHLH* genes, *cis*-elements were analyzed using 1500 bp upstream sequences of the promoter region through PlantCARE [50]. Various *cis*-elements involved in abiotic stress responses were identified, and they could be mainly divided into two groups. One is elements responsive to hormones, including abscisic acid (ABRE), jasmonic acid (CGTCA and TGACG motif), auxin (AuxRR-core and TGA-element), gibberellins (GARE motif, TATC-box, and P-box), and salicylic acid (SARE and TCA-elements). The other one is responsive to environmental stress, including low-temperature responsive elements (LTR), anaerobic induction elements (ARE), MYB binding sites involved in drought-inducibility (MBS), and defense and stress responsive elements (TC-rich repeats) (Figure S3, Table S4). All *HvbHLH* genes had light-responsive *cis*-elements, belonging to the most commonly predicted *cis*-elements in their promoters, and 98 (95.15%) *HvbHLHs* contained G-box elements. Meanwhile, there were 20 *HvbHLHs* in which the promoters included defense and stress-responsive elements (Table S4). The diversity of *cis*-elements related to stress response in *HvbHLH* promoters indicated a great difference in their functions. In addition, most genes had more than one kind of *cis*-element, suggesting that both groups could respond to multiple stresses.

### 2.7. Expression Profiles of HvbHLHs under Low Nitrogen Stress

**Figure 5:**
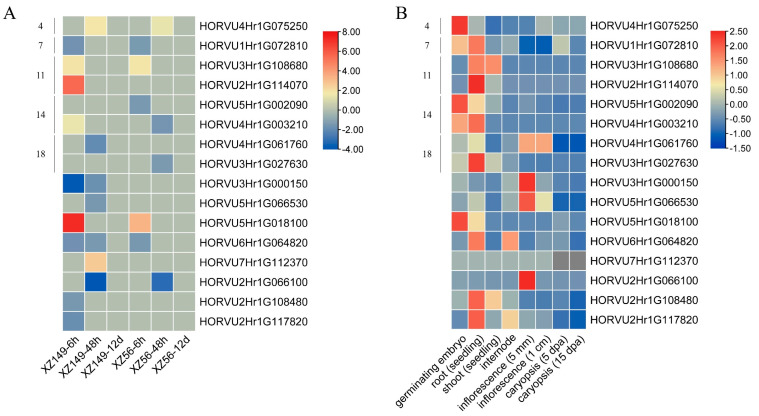
Expression profiles of the *HvbHLH* DEGs. (**A**) Expression profiles of the *HvbHLH* genes from the transcriptome in XZ149 and XZ56 at 6 h, 48 h, and 12 d under LN stress. Expression data were the values of FPKM for the LN-stressed samples divided by those of the control samples at each time point. (**B**) Expression profiles (FPKM) of the *HvbHLH* genes in different tissues of barley cultivar Morex. The color scale represents relative expression levels from high (red) to low (blue). The numbers on the left of each heat map were the subgroups to which the corresponding DEGs belong, and those of DEGs with incomplete protein sequences in the barley genome database were not labeled.

Many *HvbHLH* genes were potentially responsive to nutrition stress based on the phylogenetic and *cis*-element analyses. To verify the prediction, the expression profiles of *HvbHLHs* under LN stress were taken from transcriptome data in our previous studies (Tables S5 and S6). The transcriptome analysis was carried out using the roots of the two barley genotypes (XZ149, LN-tolerant, and XZ56, LN-sensitive) at 6 h, 48 h, and 12 d after LN stress by RNA-seq [35,51]. Consequently, 16 differentially expressed genes (DEGs) encoding HvbHLH proteins were identified using pair-wise comparison for each genotype under LN stress (Figure 5A and Table S7). Remarkably, no DEG was found between the two genotypes at 12 d, suggesting *HvbHLHs* may play a rapid regulatory role in response to LN stress. Furthermore, all the DEGs were changed only at one time point, except MLOC_74557 (HORVU6Hr1G064820) and MLOC_21066 (HORVU3Hr1G000150), which were down-regulated in XZ149 both at 6 h and 48 h after stress (Figure 5A).

Ten DEGs were down-regulated in their expression under LN stress, and their response patterns showed a marked difference (Figure 5A and Table S6). Six DEGs responded quickly to LN stress (at 6 h), while the other four DEGs were changed 48 h after LN stress. Five DEGs were up-regulated under LN stress, and three of them were changed at 6 h, as were the other two at 48 h. Notably, the change fold of all the up-regulated DEGs was larger in the tolerant genotype XZ149 relative to the sensitive genotype XZ56 (Figure 5A).

To further understand the functions of these DEGs, the expression patterns of *HvbHLHs* in eight plant tissues of the barley cultivar ‘Morex’ were analyzed based on the available transcriptomic data (Table S8) [52]. The expression level of these 16 *HvbHLH* DEGs was obtained in all eight tissues, and most of them showed higher expression in roots (Figure 5B).

The dynamic expression patterns of six *HvbHLH* DEGs were carried out in root at eight time points after LN stress using real-time PCR. As expected, all six of these genes responded to LN stress (Figure S4). However, different *HvbHLHs* exhibited distinct patterns of LN response, suggesting that *HvbHLHs* may be involved in the regulation of LN tolerance in different ways. For instance, the expression of MLOC_72280 (HORVU2Hr1G114070) maintained a higher level in XZ149 compared with XZ56 within 2 d under LN stress (Figure S4), while the expression of MLOC_37666 (HORVU2Hr1G108480) was down-regulated within 2 d after exposure to LN stress in XZ149 but up-regulated at 1 h and 3 h under LN stress in XZ56 (Figure S4).

### 2.8. HvbHLH56-Enhanced Tolerance to Low Nitrogen Stress in Transgenic Arabidopsis

Expression of HORVU2Hr1G114070 was significantly up-regulated in LN-tolerant genotype XZ149 but down-regulated in LN-sensitive genotype XZ56 under LN stress (Figure 5A). In light of the *cis*-elements and function prediction, combined with the expression patterns by RNA-seq and real-time PCR, HORVU2Hr1G114070 was chosen as a candidate gene for the function analysis in response to LN stress. This gene was renamed *HvbHLH56* based on *OsbHLH056* in the same subgroup (Figure 4). The transgenic Arabidopsis plants overexpressing *HvbHLH56* cloned from XZ149 were generated. Three independent homozygous transgenic lines, OE-1, OE-2, and OE-3, with relatively high expression levels of *HvbHLH56* (Figure S5), were employed for the further analysis.

**Figure 6:**
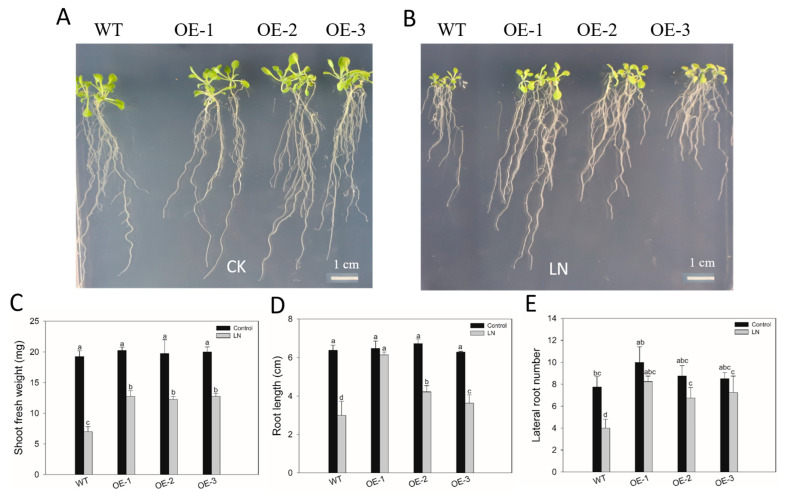
*HvbHLH56* transgenic Arabidopsis showed enhanced low nitrogen tolerance. (**A**,**B**) WT and three homozygous transgenic plants grown under control and low nitrogen conditions. (**C**–**E**) Shoot fresh weight, root length, and lateral number under low nitrogen stress, respectively. Four biological replications were performed. Different lowercase letters indicated a significant difference between treatments and genotypes at *p* < 0.05.

Subsequently, the LN tolerance of *HvbHLH56* transgenic Arabidopsis plants was assessed. The 1-week-old seedlings of *HvbHLH56* transgenic lines and WT plants were treated with nutrition medium containing 1 mM NO_3_^−^ (10 mM NO_3_^−^ as a control). One week later, the growth of all the plants was inhibited by LN stress (Figure 6A,B). However, the three transgenic lines exhibited better growth compared to WT under LN stress, while there was no difference in phenotype between WT and transgenic plants under control (Figure 6A,B). The decrease in shoot fresh weight and root length in transgenic plants was less than that in WT under LN stress (Figure 6C,D). The lateral root number in the transgenic lines showed no marked difference between control and LN stress, while it was significantly decreased in WT under LN stress (Figure 6E). These results indicated that *HvbHLH56* transgenic Arabidopsis were more tolerant to LN stress than WT.

## 3. Discussion

The bHLH TFs in higher plants comprise a large family, involving plant growth, development, and stress responses [2]. It is well documented that bHLHs respond to many abiotic stresses such as cold, drought, and salt stress [19], as well as nutrient stresses such as iron, Pi, and N deficiency [25,26,27,28,29,30,31,32]. Therefore, it is imperative to make a comprehensive analysis of the bHLH TF family. N deficiency is a common issue in agricultural production worldwide. Some studies found that bHLHs are involved in LN stress response [30,31,32]; nevertheless, the relationship/roles of bHLH family genes in LN tolerance have not been clearly defined. In this study, genome-wide identification and characterization of the bHLH gene family in barley were carried out. Furthermore, the expression profiles of *HvbHLHs* under LN stress were determined for two barley genotypes differing largely in LN tolerance. Finally, the function of the gene *HvbHLH56,* which responds to LN stress, was analyzed in Arabidopsis.

As a result, 103 *HvbHLH* genes were identified in barley through genome-wide analysis (Table S1). Multiple sequence analyses further confirmed the reliability of the *HvbHLH* genes found herein, as the bHLH domain was present in all 103 HvbHLH proteins (Figure 1). These *HvbHLH* genes were distributed over the barley genome, mainly on both ends of the chromosomes (Figure 3), similar to the distribution of Arabidopsis and rice bHLH genes on their chromosomes [6,7]. Based on phylogenetic analysis, HvbHLH proteins could be classified into 20 subfamilies, which was further supported by motif and gene structure analysis (Figure 2). Members of the same subfamily showed similar gene structure and motif composition (Figure 2), indicating their similar evolutionary origins and biological functions [45]. Gene duplication occurred in the *HvbHLH* family (Figure 3), with each pair of duplicated genes derived from the same subfamily (Figure 2 and Figure 3). On the other hand, the two members of the duplication pairs may change in their expression patterns; for instance, the segmental duplication pairs HORVU4Hr1G003210 and HORVU5Hr1G002090 were differentially expressed in tissues and in their response to LN stress (Figure 5). This illuminated the possibility that the expansion of *HvbHLHs* may be accompanied by potential sub-functionalization and neofunctionalization.

Numerous bHLH genes have been systematically functionally studied in Arabidopsis and rice [6,7]. The possible function of HvbHLHs was predicted based on the evolutionary relationships between HvbHLHs and bHLHs in Arabidopsis and rice, coupled with several bHLHs related to stress responses in other plants. Almost all the members in subgroup 11 (Figure 4), including *AtbHLH100*, *AtbHLH101*, *AtbHLH38*, and *AtbHLH39* in Arabidopsis, coupled with *OsbHLH056/OsIRO2* in rice and *GmbHLH300* in soybean, played important roles in maintaining iron homeostasis under Fe deficiency [19,28,53]. This result showed that the members of the same subgroup shared similar roles, confirming the reliability of the function prediction based on phylogenetic analysis. Meanwhile, in another subgroup, *AtbHLH104*, *AtbHLH105*, *AtbHLH115,* and *AtbHLH34* in Arabidopsis, as well as *OsbHLH058* and *OsbHLH059,* positively regulate iron deficiency responses [19,54]. Thus, the HvbHLHs in subgroups 11 and 7 may play roles in iron deficiency responses. On the other hand, HvbHLHs sharing similar roles, even those in the same subgroup, displayed diverse expression patterns (Figure 5), indicating that they may perform the same function in different ways.

In addition, members of the same subgroup also exhibited multiple roles in stress responses. For instance, in subgroup 17, *VAICE1* and *VAICE2* in wild grape could regulate cold tolerance, while *PebHLH35* in *Populus euphratica* confers drought tolerance [20,49]. Furthermore, many bHLHs have been shown to perform multiple functions in response to stress, such as *TabHLH1* [31]. This indicated that their functions may have been differentiated in the same subgroup or that one bHLH gene could respond to multiple stresses. Diverse kinds of stress-related *cis*-elements discovered in promoter regions of *HvbHLHs* may also suggest that *HvbHLHs* have the potential to play roles in diverse stressors.

Consequently, many *HvbHLHs* were considered to play roles in nutrition deficiency stress, such as iron and N deficiency responses [32,54] in subgroups 11 and 25, respectively. To further explore the possible functions of the bHLH family in LN tolerance, the expression profiles of *HvbHLH* genes were analyzed using two barley genotypes differing in LN tolerance. A total of 16 DEGs encoding HvbHLH proteins were identified in the two genotypes after 6 h and 48 h treatments (Figure 5A). Unexpectedly, no DEG was found among *HvbHLHs* in subgroup 25 [32]. Notably, the change fold of six up-regulated DEGs was much higher in the LN-tolerant genotype XZ149 than in the LN-sensitive genotype XZ56 under LN stress (Figure 5A). In particular, MLOC_72280 (HORVU2Hr1G114070) and MLOC_42946 (HORVU4Hr1G003210) were up-regulated in XZ149 but not changed or down-regulated in XZ56 (Figure 5A). It is worthy to determine the roles of these *HvbHLH* genes in XZ149. As HORVU2Hr1G114070 may be associated with Fe deficiency response [19], and there was cross-talking regulation among nutrition metabolism [35]. Thus, the function of *HvbHLH56* (HORVU2Hr1G114070) was analyzed in Arabidopsis. At last, transgenic Arabidopsis overexpressing *HvbHLH56* cloned from XZ149 was shown to enhance its tolerance to LN stress (Figure 6). It has been reported that overexpression of *TabHLH1* in tobacco improved plant tolerance to N deprivation via regulation of nitrate transporter (NRT) gene transcription [31]. In Arabidopsis, the expression of specific bHLH transcription factors was enhanced in the presence of N deficiency, accompanied by anthocyanin accumulation [55,56]. In future studies, we may focus on the roles of the candidate genes in the anthocyanin synthesis pathway and the transcriptional regulation of NRT genes under LN stress.

## 4. Materials and Methods

### 4.1. Identification of bHLH Genes in Barley

The protein sequences of bHLH in *Arabidopsis thaliana* and *Oryza sativa* were retrieved from TAIR (http://www.arabidopsis.org (accessed on 7 December 2022)) and RGAP (http://rice.plantbiology.msu.edu/ (accessed on 7 December 2022)), respectively. Barley sequence data were downloaded from IPK (https://webblast.ipk-gatersleben.de/barley_ibsc/ (accessed on 1 June 2020)) and Gramene (http://ensembl.gramene.org/Hordeum_vulgare/Info/Index (accessed on 1 June 2020)). Totally 329 *Arabidopsis* and rice bHLH protein sequences (listed in Table S9) were used to search against the barley protein sequences using BlastP with a threshold of an e-value < 1e^−10^ [36,37]. Moreover, the predicted HvbHLH proteins were also searched against the barley protein database employing the HMMER program with Hidden Markov Model (HMM) profiles of the bHLH domain (PF00010), obtained from the Pfam database (http://pfam.xfam.org/ (accessed on 7 December 2022)) [38]. After removing redundant and incomplete protein sequences, the confirmation of the predicted bHLH proteins was performed through the Pfam 35.0 online software (http://pfam.sanger.ac.uk/search (accessed on 10 January 2023)) and the NCBI Conserved Domain Search (http://www.ncbi.nlm.nih.gov/Structure/cdd/wrpsb.cgi (accessed on 10 January 2023)) [39,40]. The physiochemical properties of bHLH proteins were calculated using ExPASy (https://web.expasy.org/protparam/ (accessed on 26 January 2023)) [57]. The subcellular localization of the HvbHLHs was predicted through the online software WoLF PSORT (https://wolfpsort.hgc.jp/ (accessed on 28 March 2023)) [41].

### 4.2. Chromosomal Location and Gene Duplication

The distribution of *HvbHLH* transcription factors on chromosomes was mapped by TBtools 1.120 [43], according to the specific positions of barley genes obtained from IPK. The segmental and tandem duplications were identified by MCScanX with default settings in TBtools 1.120 [43]. The synteny relationships of *HvbHLHs* were also displayed using TBtools 1.120 [43].

### 4.3. Phylogenetic Analysis

At first, all the 103 *HvbHLHs,* 162 *AtbHLHs,* and 167 *OsbHLHs*, coupled with 7 *bHLHs* known to be involved in abiotic stress responses in other plants, were used for the phylogenetic analysis. Sixty-five genes from Arabidopsis and rice were classified into subgroups without *HvbHLHs*; thus, these genes were not employed in the tree we finally constructed. Multiple alignments were performed based on the sequences of bHLHs in barley, *Arabidopsis thaliana*, rice, and other plants using ClustralW (http://www.genome.jp/tools/clustalw/ (accessed on 28 March 2023)) [58]. The unrooted phylogenetic tree was constructed by MEGA 10.1.8 software employing the neighbor-joining (NJ) method and bootstrapping with 1000 replicates.

### 4.4. Exon/Intron Structure and Motif Analysis

The exon/intron structure of the barley bHLH genes was determined by alignment of the cDNAs and their corresponding genomic DNA sequences from the barley genome database. The conserved motifs were predicted on the online MEME 5.5.1 tool (https://meme-suite.org (accessed on 8 March 2023)) [44]. A diagrammatic sketch structure and the motif composition of HvbHLHs were mapped through TBtools 1.120 software [43].

### 4.5. Cis-Elements in Promoter Regions of HvbHLHs

To predict the *cis*-elements in the promoter regions of HvbHLHs, the 1500 bp upstream sequences from the start codon of each HvbHLH were retrieved. The cis-element distribution was then investigated in PlantCARE (http://bioinformatics.psb.ugent.be/webtools/plantcare/html/ (accessed on 8 March 2023)) [50]. The stress-related *cis*-elements were mapped in each promoter region through TBtools 1.120 software [43].

### 4.6. Plant Materials and Stress Treatments

Two Tibetan wild barley genotypes, XZ149 (LN-tolerant) and XZ56 (LN-sensitive), were used for transcriptome and real-time PCR. The barley seedlings were cultivated in hydroponics in a plant growth chamber (22/18 °C, day/night), according to Quan et al. (2016) [35]. The components of the culture solution were as follows: 2 mM NaNO_3_, 0.18 mM K_2_SO_4_, 0.18 mM KH_2_PO_4_, 0.63 mM MgSO_4_, 0.36 mM CaCl_2_, 20.9 µM NaFe (Ⅲ)-EDTA, 4.5 µM MnCl_2_, 0.38 µM ZnSO_4_, 0.16 µM CuSO_4_, 46.9 µM H_3_BO_3_, and 0.062 µM H_2_MoO_4_. Three-leaf-stage seedlings were exposed to 0.2 mM N (LN stress) and 2 mM N (control). The roots in XZ149 and XZ56 were taken for transcriptomic analysis at 6 h, 48 h, and 12 d and for real-time PCR analysis at 1 h, 3 h, 6 h, 24 h, 48 h, 3 d, 6 d, and 12 d after LN stress.

The full-length coding sequence of *HvbHLH56* cloned in XZ149 was recombined into the pCAMBIA1300-flag vector. The recombinant vector was used for the transformation of Arabidopsis plants using the floral dipping method. Homozygous T3 transgenic Arabidopsis lines and Colombia (WT) plants were used for phenotypic analysis under LN stress. The Arabidopsis seeds were germinated and cultured for one week in vertical agar plates (16 h light/8 h dark) with a constant temperature of 25 °C and a light intensity of 100 μmol m^−2^ s ^−1^. The medium contains the following compounds: 10 mM KNO_3_, 0.5 mM CaSO4, 0.5 mM MgCl_2_, 1 mM KH_2_PO_4_, 50 μM NaFe (Ⅲ)-EDTA, 2.5 mM MES, 50 μM H₃BO₃, 0.03 μM (NH_4_) _6_Mo_7_O_24_, 12 μM MnCl_2_, 1 μM CuCl₂, 1 μM ZnCl₂, 30 g/L sucrose, and 8 g/L agar. The medium with 1 mM NO_3_^-^ was used as LN treatment, and that containing 10 mM NO_3_^−^ was used as a control. The missing K element was replaced with KCl in the medium for the LN treatment [59]. After 7 days of LN treatment, the phenotype of the plants was observed, and samples were taken for the determination of growth indexes.

### 4.7. Transcriptome Analysis of HvbHLH Genes in Response to LN Stress

Totally, 24 cDNA libraries (two genotypes (XZ149, LN-tolerant, and XZ56, LN-sensitive), two treatments (LN stress and control), three time points (6 h, 48 h, and 12 d), and two biological replications) were constructed using the Illumina TruSeq™ RNA Sample Preparation Kit (Illumina, San Diego, CA, USA) in our RNA-seq analysis. Each cDNA library was sequenced on an Illumina NextSeq 500 platform. Raw reads with 2 × 75 bp pair-ends were generated through the Illumina data processing pipeline. Furthermore, the qualified clean reads were mapped against the barley Morex genomes using TopHat 2.1.1 (http://tophat.cbcb.umd.edu/ (accessed on 24 October 2022)), and the mapping reads were employed to analyze splice junctions between exons. Meanwhile, the genomes of Arabidopsis and barley Morex were used as reference genomes for the annotation of transcriptome data.

Gene expression was calculated using FPKM (fragments per kilobase of exon per million fragment-mapped reads) [60]. Differential expression analysis between control and treatment was performed using DESeq2 [61]. The threshold for screening DEGs was set at FDR < 0.05 and FPKM ≥ 1 at least in one of the samples [62]. Each relative expression level (fold change) of *HvbHLHs* was displayed as the FPKM of LN stress divided by that of control. The fold change of *HvbHLH* DEGs was shown in the heatmaps created by TBtools 1.120 software by a color gradient from low (blue) to high (red) [43]. The datasets used and/or analyzed during the current study are available from the online websites https://doi.org/10.1186/s12870-016-0721-8 (accessed on 25 May 2023) [35] and https://doi.org/10.1186/s12870-019-1668-3 (accessed on 25 May 2023) [51].

### 4.8. The Dynamic Expression of HvbHLH Genes by Real-Time PCR in Response to LN Stress

The expression of *HvbHLH* genes in roots was analyzed by real-time PCR. RNA extraction and first-strand cDNA synthesis were performed with the FastPure Plant Total RNA Isolation Kit and Hiscript III Reverse Transcriptase (Vazyme, Nanking, China), respectively. The gene-specific primers for *HvbHLH* and the internal control gene *HvGAPDH*, designed by primer-blast (https://www.ncbi.nlm.nih.gov/tools/primer-blast/ (accessed on 12 January 2023)), were present in Table S10. The real-time PCR was analyzed on a CFX96 system (Bio-Rad, Hercules, CA, USA) with three biological replicates and three technical replicates. For relative quantification, the relative expression was set as the fold change referred to the expression under control and calculated by the comparative CT method [63].

### 4.9. Expression Pattern Analysis of HvbHLH Genes in Tissues

Transcriptomic expression levels of *HvbHLH* genes in 8 different tissues (4-day embryos dissected from germinating grains, roots, and shoots from seedlings (10 cm shoot stage), developing inflorescence (5 mm and 1–1.5 cm), the third internode at the six leaf visible stage, 5 and 15 days post-anthesis developing grain (bracts removed)) of the cultivar ‘Morex’ were obtained from EMBL-EBI (https://www.ebi.ac.uk/gxa/plant/experiments (accessed on 1 March 2023)) [52].

### 4.10. Statistical Analysis

A significant difference for physiological traits among treatments and genotypes was analyzed using the Duncan’s Multiple Range Test on SPSS 16.0 software, and the difference at *p* < 0.05 was considered significant. The bar charts were generated in SigmaPlot 10.0. The relative change of each trait was calculated by the value of LN stress/control.

## 5. Conclusions

In summary, 103 bHLH genes were identified in barley by a genome-wide analysis, and their evolutionary relationships were clarified using phylogenetic, conserved motif, and exon/intron structure analyses. The expression profiles revealed that at least 16 *HvbHLH* genes may respond to LN stress in barley. The *HvbHLH* DEGs in XZ149 may be valuable for further functional characterizations of bHLH genes under LN stress and breeding barley cultivars with LN tolerance.

## Data Availability

The datasets used and/or analyzed during the current study are available from the first author on reasonable request, and her email address is bio_quanxy@ujn.edu.cn.

## Supplementary Materials

The following supporting information can be downloaded at: https://www.mdpi.com/article/10.3390/ijms24119740/s1.

## Abbreviations

bHLH, basic helix-loop-helix; DEG, differentially expressed gene; DPA, day post anthesis; FPKM, fragments per kilobase of exon per million fragment-mapped reads; LN, low nitrogen; N, nitrogen; NRT, nitrate transporter; NUE, nitrogen use efficiency; TFs, transcription factors.
