# Supplementary material for: Genome-Wide Analysis of Barley bHLH Transcription Factors and the Functional Characterization of HvbHLH56 in Low Nitrogen Tolerance in Arabidopsis
Source: Int J Mol Sci. 2023 Jun 4;24(11):9740. doi: 10.3390/ijms24119740 (PMC10253708; doi:10.3390/ijms24119740)
Supplement: Supplementary file 1 [file ijms-24-09740-s001.zip › ijms-2349308-supplementary figures.pdf]

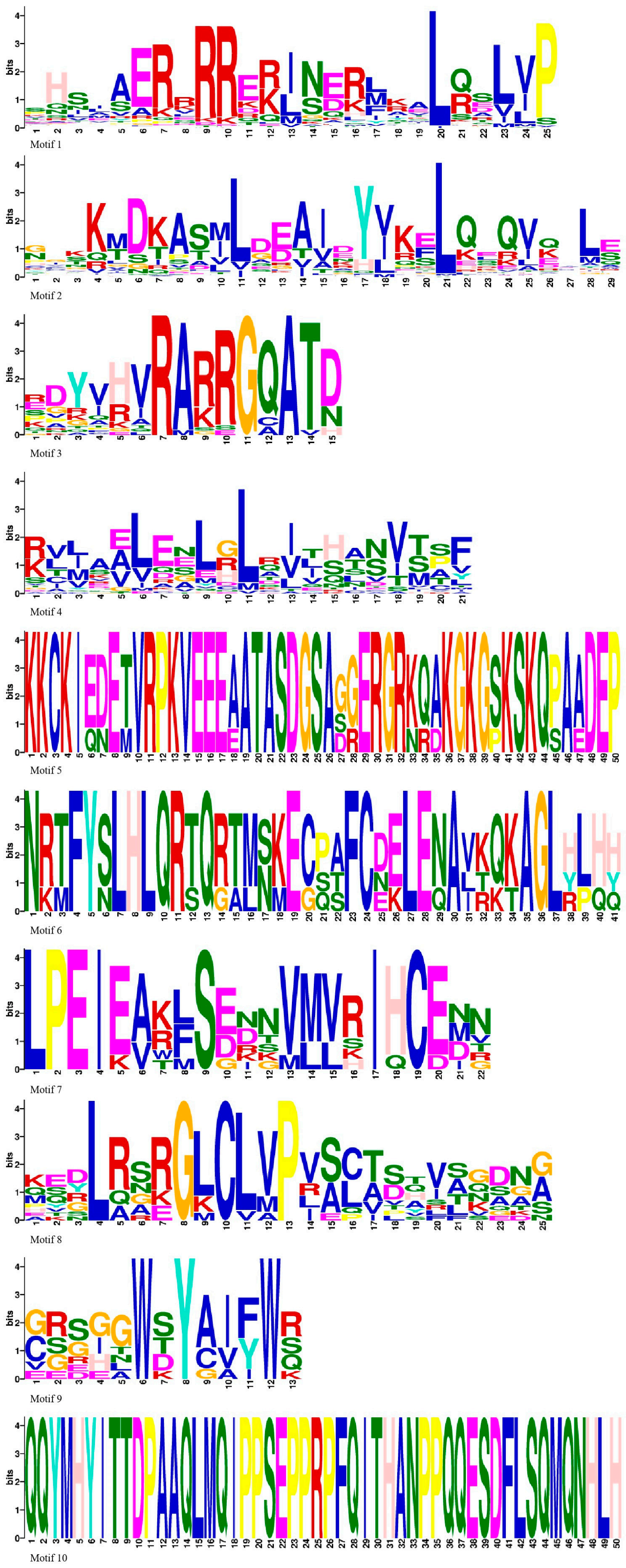

Figure S1 Conserved motifs of bHLH domains in barley

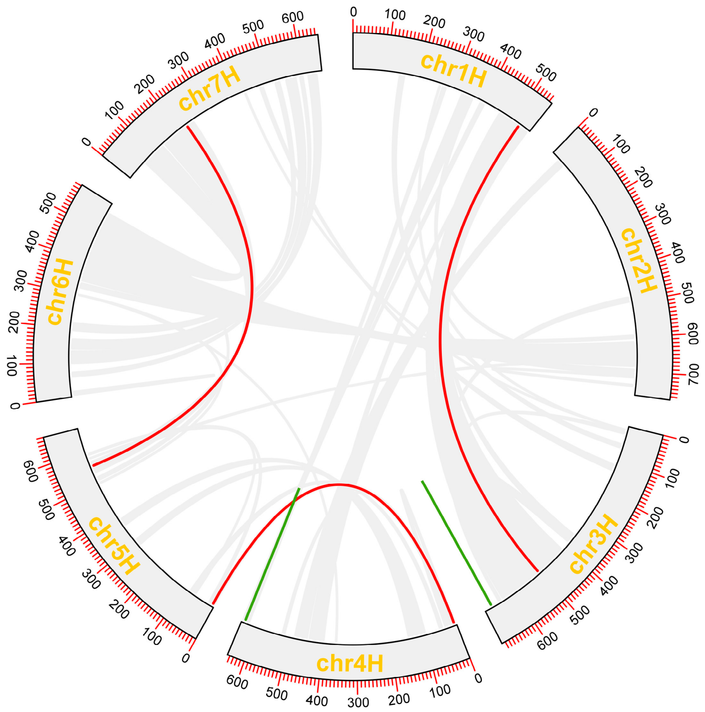

Figure S2 The synteny analysis of *HvbHLH* family in barley. Gray lines indicate all synteny blocks in the barley genome, and the red and green lines indicate segmental and tandem duplicated *HvbHLH* gene pairs, respectively. The chromosome number is indicated at the bottom of each chromosome.

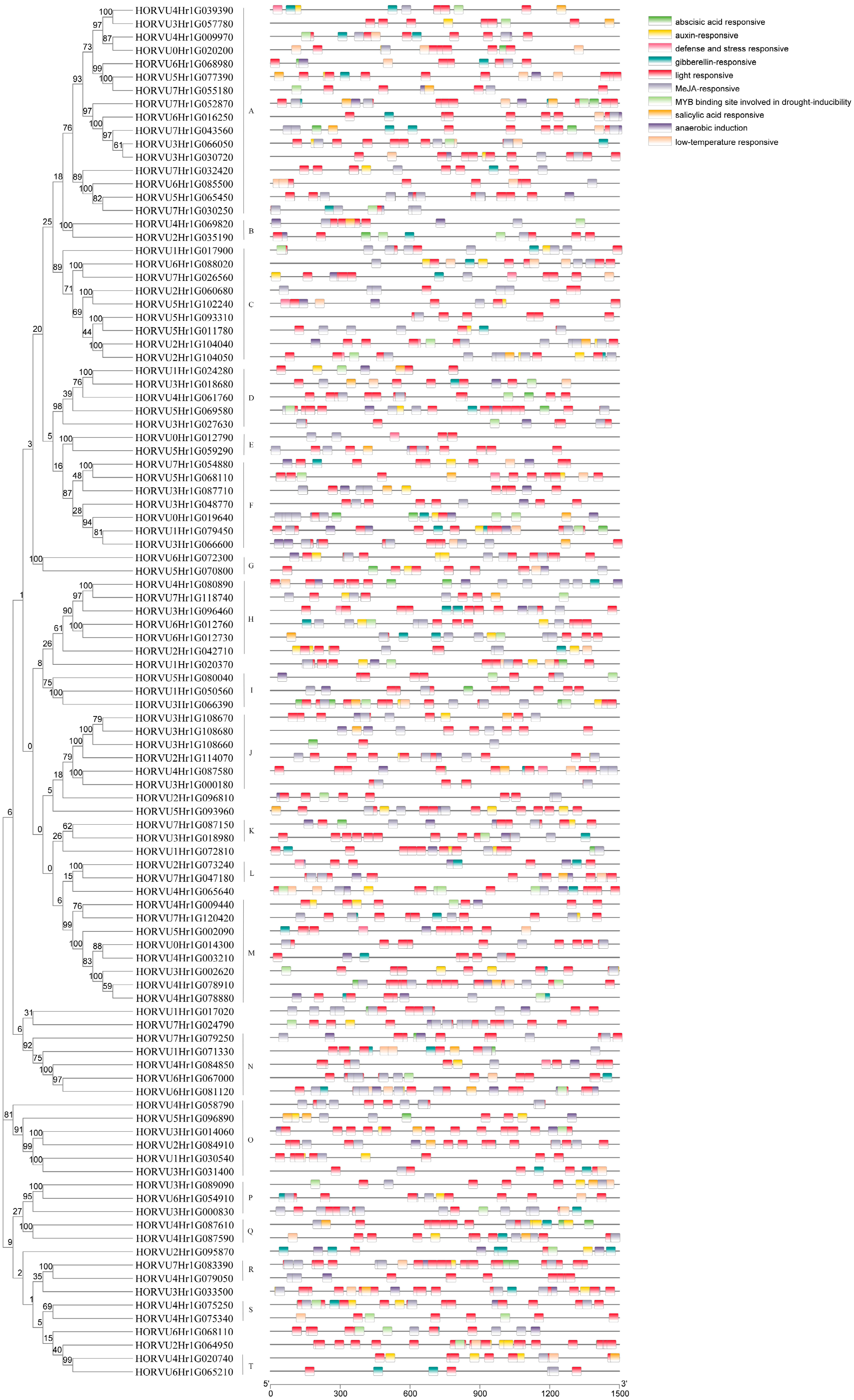
HORVU4Hr1G039390
HORVU3Hr1G057780
HORVU4Hr1G009970
HORVU0Hr1G020200
HORVU6Hr1G068980
HORVU5Hr1G077390
HORVU7Hr1G055180
HORVU7Hr1G052870
HORVU6Hr1G016250
HORVU7Hr1G043560
HORVU3Hr1G066050
HORVU3Hr1G030720
HORVU7Hr1G032420
HORVU6Hr1G085500
HORVU5Hr1G065450
HORVU7Hr1G030250
HORVU4Hr1G069820
HORVU2Hr1G035190
HORVU1Hr1G017900
HORVU6Hr1G088020
HORVU7Hr1G026560
HORVU2Hr1G060680
HORVU5Hr1G102240
HORVU5Hr1G093310
HORVU5Hr1G011780
HORVU2Hr1G104040
HORVU2Hr1G104050
HORVU1Hr1G024280
HORVU3Hr1G018680
HORVU4Hr1G061760
HORVU5Hr1G069580
HORVU3Hr1G027630
HORVU0Hr1G012790
HORVU5Hr1G059290
HORVU7Hr1G054880
HORVU5Hr1G068110
HORVU3Hr1G087710
HORVU3Hr1G048770
HORVU0Hr1G019640
HORVU1Hr1G079450
HORVU3Hr1G066600
HORVU6Hr1G072300
HORVU5Hr1G070800
HORVU4Hr1G080890
HORVU7Hr1G118740
HORVU3Hr1G096460
HORVU6Hr1G012760
HORVU6Hr1G012730
HORVU2Hr1G042710
HORVU1Hr1G020370
HORVU5Hr1G080040
HORVU1Hr1G050560
HORVU3Hr1G066390
HORVU3Hr1G108670
HORVU3Hr1G108680
HORVU3Hr1G108660
HORVU2Hr1G114070
HORVU4Hr1G087580
HORVU3Hr1G000180
HORVU2Hr1G096810
HORVU5Hr1G093960
HORVU7Hr1G087150
HORVU3Hr1G018980
HORVU1Hr1G072810
HORVU2Hr1G073240
HORVU7Hr1G047180
HORVU4Hr1G065640
HORVU4Hr1G009440
HORVU7Hr1G120420
HORVU5Hr1G002090
HORVU0Hr1G014300
HORVU4Hr1G003210
HORVU3Hr1G002620
HORVU4Hr1G078910
HORVU4Hr1G078880
HORVU1Hr1G017020
HORVU7Hr1G024790
HORVU7Hr1G079250
HORVU1Hr1G071330
HORVU4Hr1G084850
HORVU6Hr1G067000
HORVU6Hr1G081120
HORVU4Hr1G058790
HORVU5Hr1G096890
HORVU3Hr1G014060
HORVU2Hr1G084910
HORVU1Hr1G030540
HORVU3Hr1G031400
HORVU3Hr1G089090
HORVU6Hr1G054910
HORVU3Hr1G000830
HORVU4Hr1G087610
HORVU4Hr1G087590
HORVU2Hr1G095870
HORVU7Hr1G083390
HORVU4Hr1G079050
HORVU3Hr1G033500
HORVU4Hr1G075250
HORVU4Hr1G075340
HORVU6Hr1G068110
HORVU2Hr1G064950
HORVU4Hr1G020740
HORVU6Hr1G065210
A
B
C
D
E
F
G
H
I
J
K
L
M
N
O
P
Q
R
S
T
5'
3'
0
300
600
900
1200
1500
abscisic acid responsive
auxin-responsive
defense and stress responsive
gibberellin-responsive
light responsive
MeJA-responsive
MYB binding site involved in drought-inducibility
salicylic acid responsive
anaerobic induction
low-temperature responsive

Figure S3 Predicted *cis*-elements in *HvbHLH* promoters. Promoter sequences (-1500 bp) of 103 *HvbHLH* genes were analyzed by PlantCARE. The upstream length to the translation starting site can be inferred according to the scale at the bottom.

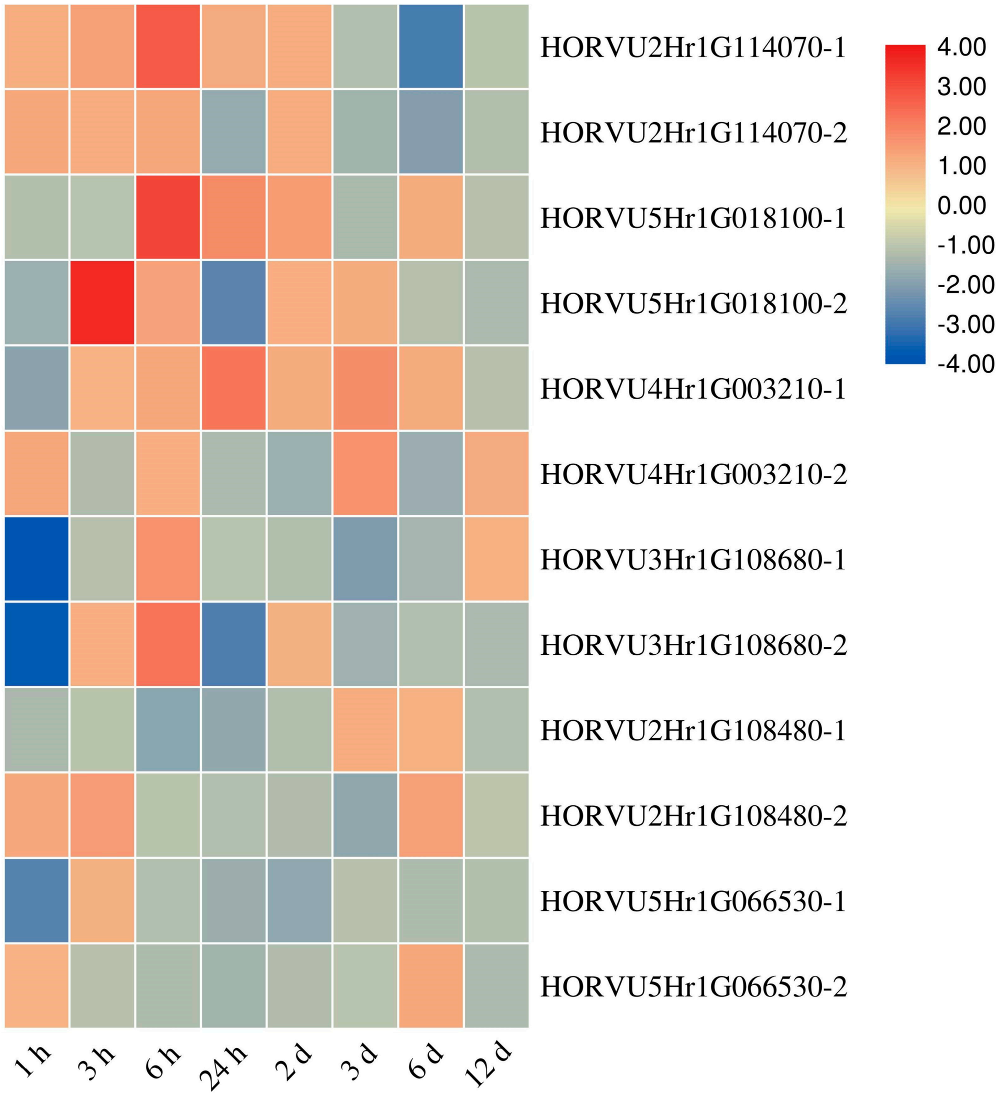

Figure S4 Expression analysis of the *HvbHLH* genes in XZ149 and XZ56 at 8 time points after LN stress. Expression data were the values of LN/control at each time point. The color scale represents relative expression levels from high (red) to low (blue).

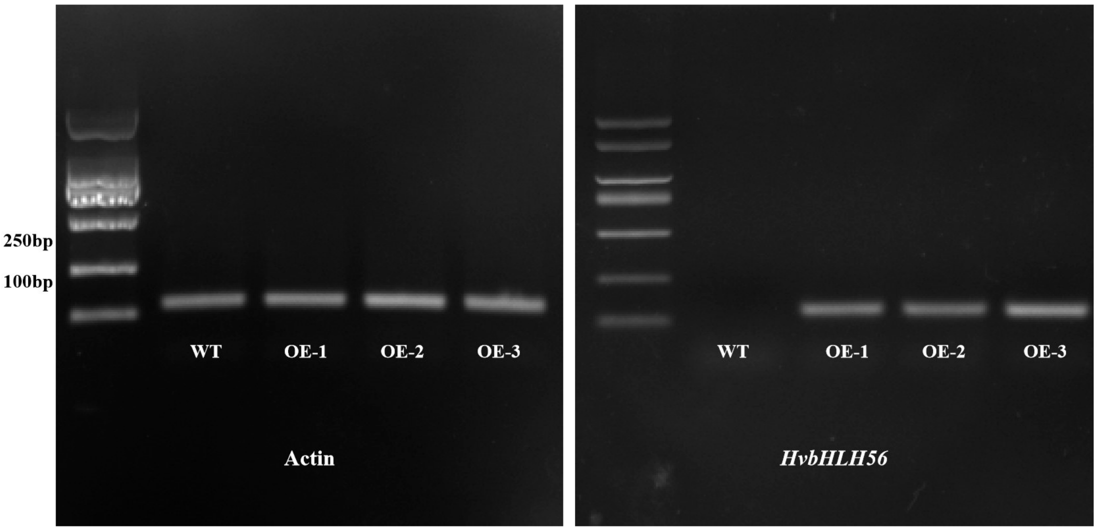

Figure S5 Relative expression of *HvbHLH56* in WT and three homozygous transgenic lines by semi-quantitative PCR. The actin8 gene was used as an internal control. The primer of actin for semi-quantitative PCR was listed in S10.
